# Progress and challenges in tuberculosis preventive treatment in the Western Pacific Region: a situational analysis of seven high tuberculosis burden countries

**DOI:** 10.1186/s41182-025-00805-6

**Published:** 2025-09-02

**Authors:** Kyung Hyun Oh, Alvin Kuo Jing Teo, Manami Yanagawa, Avinash Kanchar, Dennis Falzon, Cecily Miller, Youngeun Choi, Gyeong In Lee, Fukushi Morishita, Kalpeshsinh Rahevar, Huong Thi Giang Tran, Rajendra Prasad Hubraj Yadav, Regional TB Consortium, Anousone Sisouvanh, Bunleng Bith, Clarissa Blanca Halum, Ganpurev Byambaa, Herolyn Nindil, Huyen Thi Thanh Truong, Janet Ibay, Kim Eam Khun, Narantuya Jadambaa, Quang Hieu Vu, Sakhone Suthepmany, Serongkea Deng, Uyanga Erdenebileg, Vibol Iem, Vilath Seevisay, Walley Ambano, Zhenhong Li, Zhongdan Chen

**Affiliations:** 1 World Health Organization Regional Office for the Western Pacific, Manila, Philippines; 2 Faculty of Medicine and Health, University of Sydney, Sydney, NSW Australia; 3 The University of Sydney Infectious Diseases Institute (Sydney ID), Sydney, NSW Australia; 4 Saw Swee Hock School of Public Health, National University of Singapore and National University Health System, Singapore, Singapore; 5 World Health Organization, Global Programme on Tuberculosis and Lung Health, Geneva, Switzerland; 6 Korean Institute of Tuberculosis, Korean National Tuberculosis Association, Cheongju, Republic of Korea

**Keywords:** Tuberculosis, Tuberculosis preventive treatment, Asia and Pacific

## Abstract

**Background:**

Tuberculosis preventive treatment (TPT) can avert progression from infection to disease, yet scale-up across the World Health Organization Western Pacific Region is patchy. To guide acceleration, we assessed progress, challenges and responses in seven high-burden countries—Cambodia, China, Lao People’s Democratic Republic (PDR), Mongolia, Papua New Guinea, the Philippines and Viet Nam—drawing on 2015–2023 programme data, structured questionnaires, follow-up interviews and a regional validation workshop.

**Main body:**

Six of the seven countries have issued national TPT guidelines and five now offer shorter rifapentine- or rifampicin-based regimens. The number of people started on TPT rose sharply in most settings, driven by household contacts aged ≥ 5 years in Cambodia, Mongolia and the Philippines and by people living with HIV in Lao PDR and Papua New Guinea. However, coverage of children under five and other high-risk groups remains low. Cascade analysis revealed major attrition between screening and TPT initiation.

Key obstacles, viewed through a socio-ecological lens, include: individual complacency, fear of adverse events and limited provider confidence; stigma and consent barriers in migrant households; intermittent staff training, medicine stock-outs and weak digital tools; long journeys to health facilities; and policy–practice gaps such as the absence of child-friendly formulations and non-notification of tuberculosis infection.

Countries and partners endorsed a tiered package combining patient-centred counselling, mobile reminders, shorter paediatric regimens, stigma-reduction campaigns and remote e-consent. Health systems will reinforce staff training, digital supply-chain and adherence tools, while decentralised one-stop outreach and community health-workers extend coverage. A multisector task force will fast-track paediatric fixed-dose registration, make infection notifiable and absorb preventive treatment costs into national budgets and insurance schemes.

**Conclusions:**

The introduction of shorter regimens and rising enrolment confirm that rapid gains are achievable, yet wide disparities persist across age groups, risk categories and care-cascade stages. Implementing the agreed client, community, institutional and policy interventions—backed by integrated governance and sustainable domestic funding—can convert TPT from a promising guideline into a routine, life-saving component of primary health care throughout the Western Pacific Region.

**Supplementary Information:**

The online version contains supplementary material available at 10.1186/s41182-025-00805-6.

## Background

Tuberculosis (TB) remains a major public health challenge in the Western Pacific Region, with a sluggish annual decline in TB incidence of just 1.2% between 2015 and 2023 [1]. In 2023, the region still accounted for approximately 18% of the global TB burden, underscoring the need for intensified action to combat the disease [1].

TB preventive treatment (TPT) is a crucial intervention in the global effort to eliminate TB [2]. TPT plays a vital role in preventing the progression of TB infection to TB disease, which is essential for reducing the overall TB burden. This is particularly important for high-risk populations, such as people living with HIV, household contacts, and those with compromised immune systems. By curbing progression to TB disease, TPT may reduce the spread of TB within communities.

Since 2015, access to TPT has expanded dramatically, with millions of people initiating therapy as the World Health Organization (WHO) has broadened its guidance [1]. Key milestones include: the 2018 conditional recommendation to treat household contacts aged ≥ 5 years, adolescents and adults of bacteriologically confirmed pulmonary TB in high-incidence countries [3]; the 2020 introduction of two short-course alternatives—one month of daily rifapentine plus isoniazid and four months of daily rifampicin [4]; the 2022 conditional endorsement of *Mycobacterium tuberculosis* antigen-based skin tests for diagnosing latent infection [5]; and, most recently, the 2024 strong recommendation for six months of daily levofloxacin for contacts exposed to multidrug- or rifampicin-resistant TB [2].

However, implementation challenges persist, including gaps in identifying eligible individuals, logistical barriers in drug delivery and adherence support, and inadequate integration of TPT services into broader health systems. Moreover, insufficient funding and varying levels of political commitment in different countries hinder consistent implementation [6]. The COVID-19 pandemic has exacerbated these challenges by disrupting health services, redirecting resources, and causing delays in TB diagnosis and treatment, including TPT implementation [7]. These disruptions have reversed some of the progress made in TB prevention, increasing the urgency to address these setbacks.

In the Western Pacific Region, TPT is also a priority action to end TB. As the region continues its efforts towards TB elimination, understanding the progress and challenges in implementing TPT is critical for shaping effective strategies and achieving the ambitious goals set in the Western Pacific Regional Framework to end TB [8, 9]. The framework identifies 10 priority countries based on their TB burden. Among these, seven countries—Cambodia, China, Lao People’s Democratic Republic (PDR), Mongolia, Papua New Guinea, the Philippines, and Viet Nam—were selected for this review based on their absolute number of TB cases and TB incidence rates. Understanding the progress and challenges in implementing TPT in these countries is crucial for tailoring future strategies to achieve the ambitious goals set by the framework.

## Methods

We assessed progress in TPT across seven priority countries by extracting annual indicators for 2015–2023 from the WHO Global TB database [10].

In July 2023, we developed a structured questionnaire as a formatted, editable Microsoft Excel sheet to understand key domains of programmatic implementation of TPT: (1) national TPT interventions; (2) identifying populations for TPT; (3) strategies for ruling out TB disease and testing for TB infection; (4) models of care for TPT including regimens; and (5) mechanisms for drug-safety monitoring and management. The draft tool was reviewed by technical staff at the WHO Regional Office for the Western Pacific (WPRO) and WHO headquarters, revised in line with feedback, and then disseminated by WHO WPRO via e-mail to WHO country offices for completion by national TB programme focal points.

Completed questionnaires were received from all seven countries between August and September 2023. To verify and clarify submissions, WHO WPRO and WHO country office staff conducted follow-up virtual interviews with each country’s national TB programme focal points during the same period.

Subsequently, the survey findings were presented at a regional workshop on TB preventive treatment, screening, and infection prevention and control held in Seoul, Republic of Korea, on 25–27 October 2023. The meeting brought together national TB programme focal points, staff from the WHO WPRO, WHO headquarters, and WHO country offices, and partners from the Korean Institute of Tuberculosis. Building on the survey results, participants synthesised programmatic challenges and feasible solutions through facilitated group discussions, and each country team co-developed a country-specific action plan.

In the main text, recommendations are drawn primarily from the 2024 WHO consolidated guidelines on TPT [2]. As data collection (survey and workshop) occurred in 2023, we also note the recommendations that were in force at that time, wherever recommendations have since changed, to preserve fidelity to country responses. Descriptions of programmatic implementation are based on the survey and interview data, refined during the workshop. Challenges and proposed solutions generated during the Seoul workshop (via facilitated group discussions) are organised using the socio-ecological framework—individual, interpersonal, institutional, community and policy levels—with items that span multiple tiers classified as cross-cutting [11].

### National TPT interventions

Most of the priority countries have successfully established national guidelines for TPT except Lao PDR, where guidelines are yet to be formalised. In Lao PDR and Mongolia, TPT is implemented through public health institutions, whereas in the other countries, it is implemented through a combination of public health institutions and non-governmental organisations. Funding for TPT predominantly comes from the Global Fund to Fight AIDS, Tuberculosis and Malaria (Global Fund), an international financing mechanism dedicated to combating infectious diseases, including TB. While China stands out as an exception where the national government fully funds its TPT intervention, Mongolia, Papua New Guinea, and the Philippines benefit from a hybrid funding model, with financial support shared between the governments and the Global Fund.

## Eligible populations for TPT

Table 1 categorises the populations eligible for TPT across the seven priority countries. These include people living with HIV, household contacts of TB patients, and other people at risk.

**Table 1:** Eligible populations for TPT in seven priority countries of the Western Pacific Region

	Cambodia	China	Lao PDR	Mongolia	Papua New Guinea	Philippines	Viet Nam
People living with HIV							
Adults and adolescents living with HIV	Yes	Yes	Yes	Yes	Yes	Yes	Yes
Pregnant women living with HIV	No	No	Yes	Yes	Yes	Yes	Yes
Infants aged < 12 months living with HIV who are contacts of TB	Yes	No	Yes	Yes	Yes	Yes	Yes
Children aged ≥ 12 months living with HIV	Yes	Yes	Yes	Yes	Yes	Yes	Yes
Household contacts							
Children aged < 5 years who are household contacts of bacteriologically confirmed pulmonary TB	Yes	Yes	Yes	Yes	Yes	Yes	Yes
Children aged ≥ 5 years, adolescents and adults who are household contacts of bacteriologically confirmed TB	Yes	Yes	No	Yes	No	Yes	Yes
Household contacts of MDR-TB	No	Yes	No	Yes	No	No	No
Other people at risk							
People who are initiating anti-TNF treatment or on long-term immunosuppressive therapy	Yes	Yes	No	Yes	No	Yes	Yes
People who are receiving dialysis	Yes	Yes	No	Yes	No	Yes	Yes
People who are preparing for an organ transplant	Yes	Yes	No	Yes	No	Yes	Yes
People who have silicosis	Yes	No	No	Yes	No	Yes	Yes
Prisoners	Yes	Yes	No	Yes	No	Yes	Yes
Health workers	No	No	No	Yes	No	No	Yes
Immigrants from countries with high TB burden	No	No	No	Yes	No	No	Yes
Homeless people	No	No	No	Yes	No	No	No
People who use drugs	No	No	No	Yes	No	No	No
People with diabetes	No	No	No	Yes	No	No	Yes

Lao PDR; Lao People’s Democratic Republic, TB; tuberculosis, TPT; tuberculosis preventive treatment, MDR-TB; multidrug-resistant tuberculosis, TNF; tumour necrosis factor,

### People living with HIV

WHO strongly recommends TPT for adults and adolescents living with HIV, children aged 12 months and older living with HIV, and infants under 12 months old who are living with HIV and in contact with a TB patient [2]

People living with HIV eligible for TPT across all countries, with two exceptions: pregnant women living with HIV are not eligible for TPT in Cambodia and China, and infants under 12 months with HIV who are contacts of TB are not eligible for TPT in China.

### Household contacts

WHO strongly recommends TPT for children under 5 years of age who are household contacts of individuals with bacteriologically confirmed pulmonary TB, while TPT for contacts aged 5 years and older is conditionally recommended [2]. At the time of this study, TPT was conditionally recommended for high-risk household contacts of patients with multidrug-resistant TB (MDR-TB) based on individualised risk assessment and sound clinical justification [4].

All seven priority countries have included children under 5 years old who are household contacts of individuals with bacteriologically confirmed pulmonary TB as eligible for TPT, while five of the seven priority countries: Cambodia, China, Mongolia, the Philippines, and Viet Nam, include children aged 5 years and above, as well as adolescents and adults household contacts of individuals with bacteriologically confirmed TB as eligible for TPT. However, only China and Mongolia have included household contacts of MDR-TB patients, as eligible for TPT.

### Other people at risk

WHO strongly recommends TPT for individuals initiating anti-tumor necrosis factor treatment, receiving dialysis, preparing for an organ or haematological transplant, or those who have silicosis [2]. TPT is also recommended conditionally for prisoners, healthcare workers, immigrants from countries with a high TB burden, homeless people, and people who use drugs [2]. However, at the time of this review, systematic provision of TPT was not recommended by WHO for individuals with diabetes, those who engage in harmful alcohol use, tobacco smokers, or underweight individuals, due to insufficient evidence supporting its programmatic use [4].

In practice, eligibility for TPT among these high-risk groups varies across countries in the Western Pacific Region. For example, Mongolia includes all of the above groups as eligible for TPT, whereas Lao PDR and Papua New Guinea do not include any of them.

## Ruling out TB disease and testing for TB infection

Table 2 outlines the methods used to rule out TB disease and the testing for TB infection across the seven priority countries.

**Table 2:** Ruling out TB disease and testing for TB infection in seven priority countries of the Western Pacific Region

	Cambodia	China	Lao PDR	Mongolia	Papua New Guinea	Philippines	Viet Nam
Symptom-based screening	All eligible populations	All eligible populations	All eligible populations	All eligible populations	All eligible populations	All eligible populations	All eligible populations
Chest radiography	No	All eligible populations	No	All eligible populations	All eligible populations	– Adults and adolescents living with HIV except for pregnant women – Household contacts (≥ 5 years) – Other people at risk	– Household contacts (≥ 5 years) – Other people at risk
Testing for TB infection	No	TST, IGRA, and TBST for all eligible populations	No	– TST for all eligible populations – IGRA for household contacts (all ages) only	No	TST for household contacts (≥ 5 years) and other people at risk	TST and IGRA for household contacts (≥ 5 years) and other people at risk

Lao PDR; Lao People’s Democratic Republic, TB; tuberculosis, TST; tuberculin skin test, IGRA; interferon-gamma release assay, TBST; *Mycobacterium tuberculosis* antigen-based skin test

### Symptom-based screening

As per the WHO guidelines, adults, adolescents, children, and infants living with HIV should undergo screening using a clinical algorithm. If they exhibit no symptoms of TB, they should be offered TPT [2]. For HIV-negative household contacts aged 5 years and older, as well as other people at risk, the absence of TB symptoms may be used to rule out TB disease before commencing TPT [2].

In all seven priority countries, symptom-based screening is uniformly applied across all eligible populations before TPT is administered.

### Chest radiography

WHO recommends the use of chest X-ray among people living with HIV who are on antiretroviral therapy (ART), and those with no abnormal radiographic findings be provided TPT [2]. For HIV-negative household contacts aged 5 years and older, as well as other people at risk, the absence of abnormal chest X-ray findings may be used to rule out TB disease before initiating TPT [2].

Chest X-ray is provided to all eligible populations in China, Mongolia, and Papua New Guinea to rule out TB disease; it is used mainly for household contacts aged 5 years and older in the Philippines and Viet Nam; and it is not routinely available in Cambodia and Lao PDR.

### Testing for TB infection

WHO strongly recommends the use of either a tuberculin skin test (TST) or interferon-gamma release assay (IGRA) to test for TB infection [2]. WHO also recommends *Mycobacterium tuberculosis* antigen-based skin tests (TBST) to test for TB infection [2]. However, testing is not required prior to TPT initiation for people with HIV or for household contacts aged < 5 years [2].

TST is available in four of the seven priority countries—China, Mongolia, the Philippines, and Viet Nam—while IGRA is offered only in China and Mongolia. China is the sole country using TBST. Testing for TB infection is not implemented in Cambodia, Lao PDR, or Papua New Guinea.

## Models of care for TPT

Table 3 summarises how TPT is delivered across the seven priority countries, covering regimens, service delivery platforms, and monitoring and adherence support.

**Table 3:** Models of care for TPT in seven priority countries of the Western Pacific Region

	Cambodia	China	Lao PDR	Mongolia	Papua New Guinea	Philippines	Viet Nam
Regimens	– 6H for all eligible populations – 3HP for adults and adolescents living with HIV and household contacts (all ages) only – 3HR for household contacts (all ages) only	– 6H for all eligible populations except for children aged ≥ 12 months living with HIV – 3HP for children aged ≥ 12 months living with HIV, household contacts (≥ 5 years) and other people at risk – 3HR and 4R for household contacts (all ages) and other people at risk	6H for all eligible populations	– 6H and 3HR for all eligible populations except for household contacts of MDR-TB – 1HP for adolescents living with HIV and household contacts (≥ 5 years) – Levofloxacin for household contacts of MDR-TB	6H for all eligible populations	– 6H for all eligible populations – 3HP for adults and adolescents living with HIV, children aged ≥ 12 months living with HIV, household contacts (≥ 5 years) and other people at risk – 3HR for adults and adolescents living with HIV including pregnant women, infants aged < 12 months living with HIV who are contacts of TB, household contacts (all ages) and other people at risk - 4R for adults and adolescents living with HIV	– 6H for all eligible populations – 3HP for all eligible populations aged ≥ 2 years except for pregnant women – 3HR for household contacts (all ages) and other people at risk
Service delivery platforms	– HIV clinic for adults and adolescents living with HIV – Primary care level for the other eligible populations	– Prison for prisoners – Community and primary care level for the other eligible populations	Primary care level for all eligible populations	– Secondary care level for adults and adolescents living with HIV – Primary care level for the other eligible populations	Primary care level for all eligible populations	– Prison for prisoners – Primary care level for the other eligible populations	– Prison for prisoners – Secondary care level for the other eligible populations
Monitoring and adherence support	– Self-reporting – Follow-up at health facilities – Community visits	– Self-reporting – Follow-up at health facilities – Community visits – VST	– Self-reporting - Follow-up at health facilities – Community visits	– Self-reporting - Follow-up at health facilities – Community visits – VST	Self-reporting	– Self-reporting – Follow-up at health facilities – Community visits – VST	– Self-reporting – Pill counts – Follow-up at health facilities – Community visits

Lao PDR; Lao People’s Democratic Republic, TPT; tuberculosis preventive treatment, MDR-TB; multidrug-resistant tuberculosis, 1HP; one month of daily rifapentine plus isoniazid, 3HP; three months of weekly rifapentine plus isoniazid, 3HR; three months of daily rifampicin plus isoniazid, 4R; four months of daily rifampicin monotherapy, 6H; six months of daily isoniazid monotherapy, 9H; nine months of daily isoniazid monotherapy, VST; video-supported treatment

### Regimens

WHO strongly recommends different TPT regimen options, namely 6 or 9 months of daily isoniazid (6H or 9H), or 3 months of weekly rifapentine plus isoniazid (3HP), or 3 months of daily isoniazid plus rifampicin (3HR), regardless of HIV status [2]. Additionally, alternative regimens such as 1-month of daily rifapentine plus isoniazid (1HP) or 4 months of daily rifampicin alone (4R) are also recommended [2]. For contacts exposed to MDR-TB or rifampicin-resistant TB, 6 months of daily levofloxacin is strongly recommended [2]. At the time of this study, TPT for high-risk household contacts of patients with MDR-TB was conditionally recommended without a specified regimen [4].

Across the seven countries, regimen availability is as follows: 6H is available in all; 3HR in five (Cambodia, China, Mongolia, the Philippines and Viet Nam); 3HP in four (Cambodia, China, the Philippines and Viet Nam); 4R only in China and the Philippines; and both 1HP and six months of daily levofloxacin only in Mongolia.

### Service delivery platforms

Most countries deliver TPT primarily through the primary care level, with notable exceptions. Viet Nam mainly provides TPT at secondary care facilities, while China also delivers services at community level. For people living with HIV, TPT is provided through HIV clinics in Cambodia and through secondary care in Mongolia. Prison-based delivery is implemented in China, the Philippines and Viet Nam.

### Monitoring and adherence support

All seven countries use patient self-report. Facility-based follow-up visits operate in Lao PDR, Viet Nam, China, Mongolia and the Philippines, and community-based follow-up (including home visits and local engagement) is also used in these same countries. Viet Nam incorporates pill counts into monitoring, and China, Mongolia and the Philippines use video-supported treatment to support adherence.

## Drug-safety monitoring and management

Routine clinical assessment during follow-up is conducted in Cambodia, China, Mongolia, the Philippines and Viet Nam, where suspected adverse events are managed within standard care pathways and referred according to national protocols where indicated. Formal adverse drug reaction reporting operates through national pharmacovigilance systems in Papua New Guinea and the Philippines, although these systems are not TB-specific. Systematic, regimen-specific recording of adverse events for shorter TPT regimens is not consistently in place across countries, and no programme provides aggregated national safety data disaggregated by regimen; consequently, cross-country comparisons of safety signals are not feasible.

## Progress

TPT has been scaled up in the seven priority countries in line with the WHO recommendations.

### Trends of TPT by target group

**Fig. 1:**
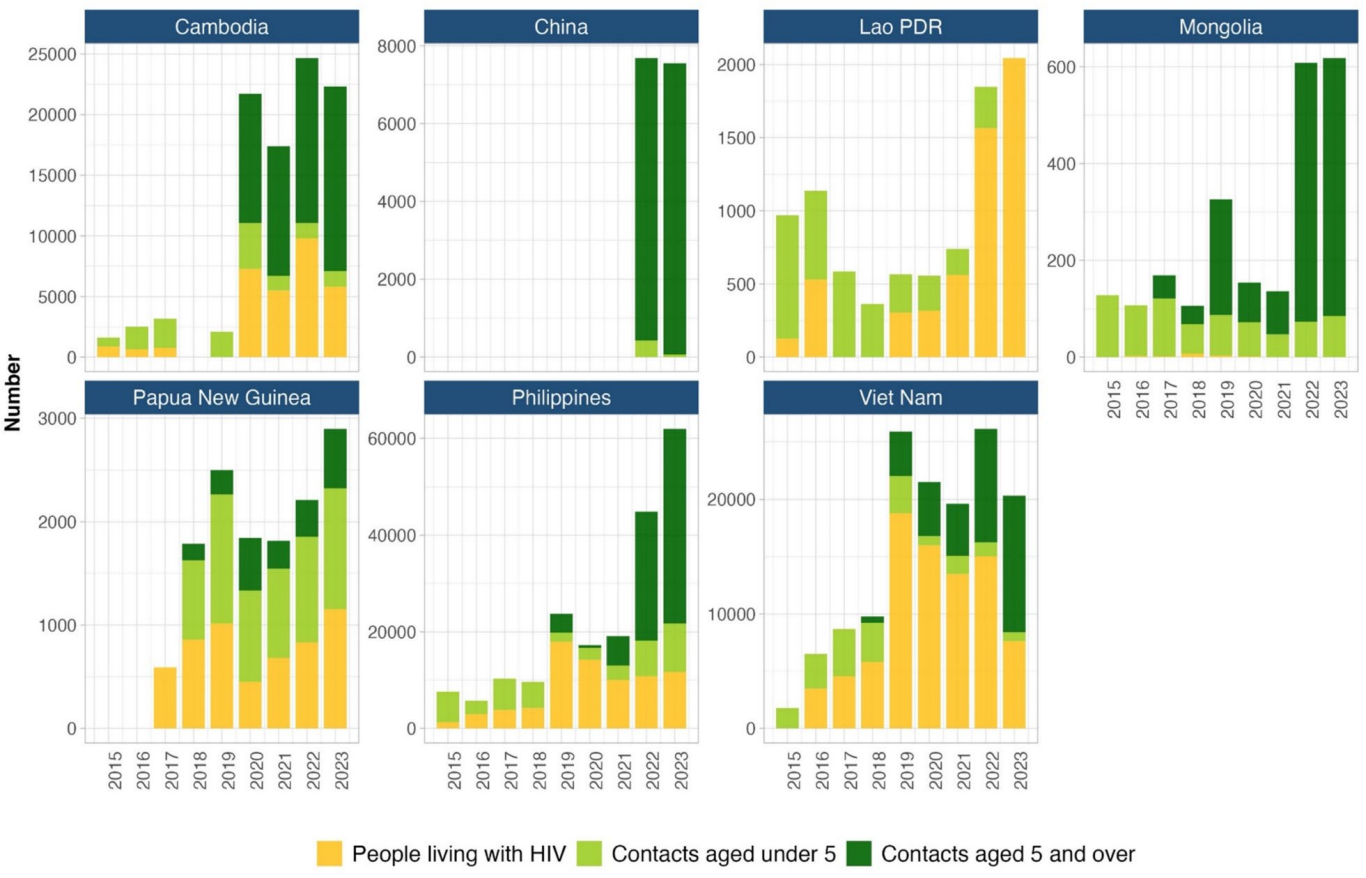
Number of tuberculosis preventive treatment by target group in seven priority countries of the Western Pacific Region, 2015–2023

Figure 1 depicts the number of individuals receiving TPT across different target groups from 2015 to 2023 in the seven priority countries. In Cambodia, a significant scale-up in TPT implementation is observed from 2020 onward, with notable contributions from all three target groups, particularly household contacts aged 5 years and over. China demonstrated steady implementation starting in 2022, focusing primarily on household contacts aged 5 years and over. Lao PDR showed gradual progress, especially among people living with HIV, with numbers peaking in 2022 and 2023. Mongolia experienced steady progress between 2015 and 2019, followed by a decline in 2020 and 2021. However, a sharp rise occurred in 2022, particularly among household contacts aged 5 years and over, with continued growth into 2023. In Papua New Guinea, gradual improvement in TPT provision was observed, particularly among children under 5 and people living with HIV. The Philippines displayed steady progress from 2015 to 2019, followed by a reduction in 2020 and 2021. However, a dramatic increase in TPT occurred from 2022, especially among household contacts aged 5 years and over. Viet Nam significantly scaled up TPT implementation for all target groups starting in 2019, peaking in 2022.

Comparative coverage in 2023 indicates that TPT uptake among people living with HIV was highest in Lao PDR, followed by the Philippines and Viet Nam (Supplementary Fig. 1). Coverage for household contacts aged < 5 years was greatest in Papua New Guinea, with Cambodia and Mongolia close behind in 2022 (Supplementary Fig. 2). Among household contacts aged ≥ 5 years, Cambodia led, followed by Mongolia and Viet Nam in 2023 (Supplementary Fig. 3).

### Care cascade of TPT among household contacts

**Fig. 2:**
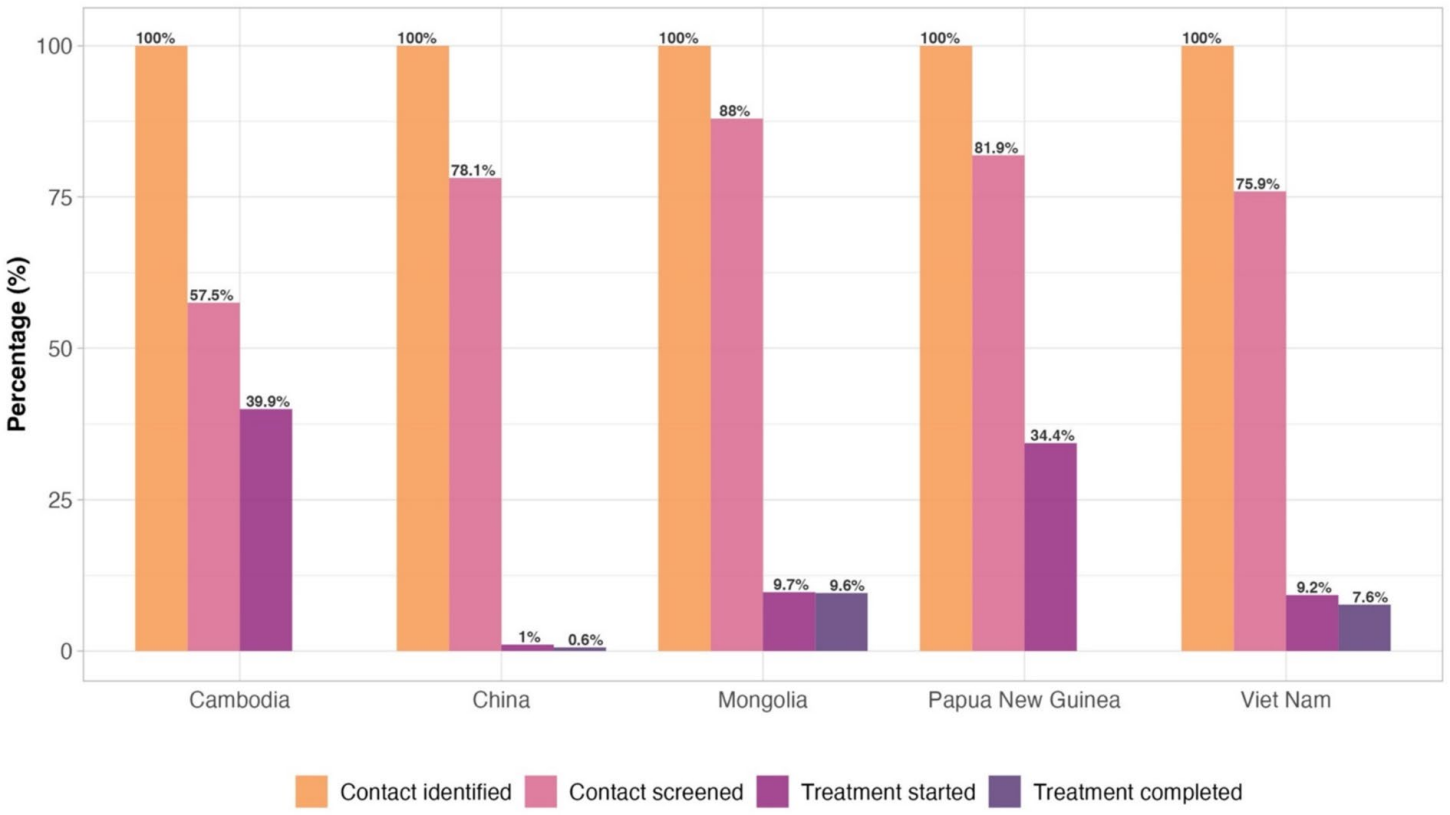
Care cascade of tuberculosis preventive treatment among all household contacts in five priority countries of the Western Pacific Region, 2021–2023

Figure 2 illustrates the TPT care cascade among household contacts in 2021–2023 across five priority countries: Cambodia, China, Mongolia, Papua New Guinea, and Viet Nam.

Among the identified contacts, Mongolia had the highest screening coverage (88%), followed by Papua New Guinea (81.9%) and China (78.1%). In contrast, Cambodia screened only 57.5% of its identified contacts. For treatment initiation among those screened, Cambodia had the highest rate (69.4%), followed by Papua New Guinea (42%), while China, Mongolia, and Viet Nam reported lower rates of 1.3%, 11%, and 12.1%, respectively. Among contacts who started treatment, Mongolia had the highest completion rate (99%), followed by Viet Nam (82.6%) and China (60%). Data on TPT completion in Cambodia and Papua New Guinea are not available.

### Use of shorter regimens for TPT

**Fig. 3:**
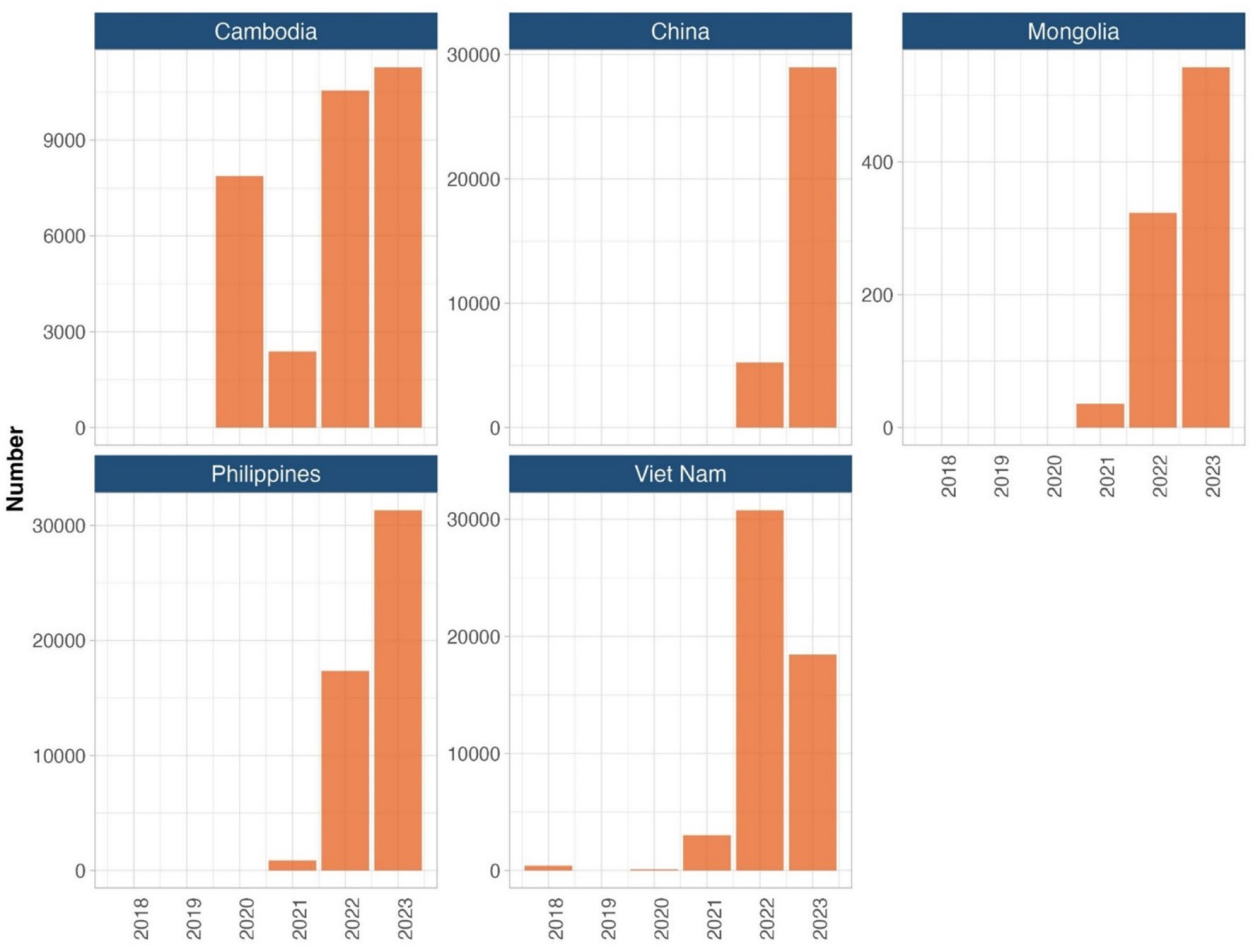
Number of tuberculosis preventive treatment using shorter regimens in five priority countries of the Western Pacific Region, 2018–2023

Figure 3 shows the number of individuals receiving TPT using shorter regimens (1HP, 3HP, 3HR, and 4R) from 2018 to 2023 across five priority countries: Cambodia, China, Mongolia, the Philippines, and Viet Nam. In Cambodia, the use of shorter TPT regimens increased significantly from 2020 to 2023, despite a notable decline in 2021, likely due to disruptions caused by the COVID-19 pandemic. China began reporting the use of shorter TPT regimens in 2022, with a sharp rise observed in 2023. Both Mongolia and the Philippines experienced a sharp increase starting in 2021. In Viet Nam, the use of shorter regimens peaked in 2022 but declined in 2023.

In 2023, shorter regimens accounted for 50% of all TPT in Cambodia (11,274/22,326), 88% in Mongolia (542/618), 51% in the Philippines (31,320/61,923) and 91% in Viet Nam (18,452/20,322). China is excluded from these proportions due to unreliable data reporting.

## Challenges

Programmatic challenges identified through the survey and interviews, and synthesised at the workshop, are described by socio-ecological level; issues spanning multiple tiers are classified as cross-cutting (Table 4) [11].

**Table 4:** Socio-ecological barriers and targeted solutions for scaling up tuberculosis preventive treatment in the Western Pacific Region

Socio-ecological level	Key challenges	Targeted solutions
Individual	• “Too-healthy” complacency • Low awareness of TPT benefits & TB risk • Fear of adverse drug reactions • Some clinicians doubt efficacy / fear resistance	• Brief risk-counselling + SMS nudges • Leaflets / chat-bots for FAQs • Short, child-friendly rifapentine regimens • Ongoing CME & bedside mentoring
Interpersonal	• TB stigma blocks disclosure & adherence • Migrant households: non-parent caregivers lack consent authority	• Peer storytelling & stigma-reduction • Secure tele-consent for parents working abroad
Institutional	• Irregular staff training • Drug / diagnostic stock-outs • High turnover erodes institutional memory • Little real-time adherence tracking	• Scheduled in-service mentoring & SOP repositories • e-logistics with buffer-stock alerts • Retention / rotation plans • Digital adherence tech (smart pill boxes, SMS)
Community	• Long travel & transport costs • Sparse facilities in rural / island areas	• Decentralise TPT to PHC, pharmacies & CHWs • “One-stop-shop” outreach (screen, CXR, pickup) • Mobile clinics / transport vouchers, guided by gap-mapping
Policy	• Narrow eligibility in some settings (e.g., exclusion of household contacts ≥ 5 years) • Guideline–practice gaps for high-risk groups (e.g., MDR-TB contacts, pregnant women with HIV) • Paediatric formulations scarce • Heavy dependence on international donors and chronic domestic underfunding • TB infection non-notifiable (no ICD-10 use) • Regulatory ambiguity on off-label preventive regimens	• Embed updated WHO advice in supervision tools • Fast-track child-friendly fixed-dose combos • Transition financing to domestic budgets/UHC with defined budget lines • Adopt ICD-10 code to make infection notifiable • Issue circulars authorising preventive regimens
Cross-cutting	• Vertical, siloed service model • Centralised care distant from communities • Policies not tailored to frontline capacity • Fragmented financing & governance	• Integrate TPT into HIV, ANC, diabetes & routine PHC • Broaden decentralised delivery • Context-specific guideline-adaptation workshops • Multi-sector task force for aligned financing, procurement & monitoring

TPT; tuberculosis preventive treatment, TB; tuberculosis, SMS; short message service, CME; continuing medical education, SOP; standard operating procedure, PHC; primary health care, CHW; community health worker, CXR; chest X-ray, UHC; universal health coverage, ANC; antenatal care, ICD-10; International Classification of Diseases, 10th Revision, MDR-TB; multidrug-resistant tuberculosis, HIV; human immunodeficiency virus, WHO; World Health Organization

### Individual level

Many eligible individuals perceive themselves as healthy and asymptomatic, leading to low motivation to initiate or adhere to TPT [12]. This issue is exacerbated by a lack of awareness regarding the benefits of TPT and an underestimation of their risk of developing active TB [13]. Concerns about potential adverse drug reactions further deter individuals from starting treatment [14]. Additionally, health care providers may contribute to these challenges due to insufficient knowledge or scepticism about the benefits of TPT [15]. Fears of contributing to drug resistance also discourage some providers from actively promoting TPT.

### Interpersonal level

Stigma associated with TB remains a significant barrier, discouraging individuals from seeking or adhering to TPT out of fear of social ostracism. In households where parents migrate for work, caregiving responsibilities often fall on extended family members or non-parental guardians who may lack the authority to make medical decisions for children, creating gaps in treatment initiation and adherence [12].

### Institutional level

Inadequate training and capacity-building for TB programme staff result in inconsistent programme delivery and suboptimal management of TPT [16]. Frequent drug stock-outs disrupt continuity of care, and high staff turnover exacerbates these issues, limiting institutional memory and sustained programme execution [15]. Furthermore, limited infrastructure and resources hinder the ability to monitor and support TPT adherence effectively [14].

### Community level

Logistical barriers, such as difficulties in accessing follow-up appointments, obtaining prescription refills, and engaging with health care services, pose significant challenges to TPT adherence [12, 15]. These issues are often compounded by systemic barriers, such as transportation challenges and health care facility shortages in remote areas, which further limit consistent engagement with TPT programmes [17].

### Policy level

Eligibility definitions remain narrow in some countries (e.g., exclusion of household contacts aged ≥ 5 years in Lao PDR and Papua New Guinea), slowing scale-up. Implementation gaps persist where policy-eligible groups are not reached in practice (e.g., pregnant women living with HIV in Mongolia; household contacts of people with MDR-TB in China). Limited child-friendly formulations constrain paediatric delivery. Heavy dependence on international donors undermines sustainability and long-term planning, while chronic domestic underfunding restricts expansion. In many settings, TB infection is non-notifiable because International Classification of Diseases-10 (ICD-10) coding has not been adopted, weakening surveillance and accountability. Regulatory uncertainty—especially around off-label preventive regimens—and fragmented pharmacovigilance add administrative burden and dampen provider uptake.

### Cross-cutting issues

TPT often operates as a vertical, add-on service rather than an integrated component of primary care. Centralised service delivery keeps prevention remote from many communities, and governance is fragmented—financing, procurement and civil-society oversight proceed on separate tracks, weakening accountability and coordination.

## Proposed solutions

Targeted interventions developed at the workshop are presented by socio-ecological level; measures that cut across multiple tiers are classified as cross-cutting (Table 4) [11].

### Individual level

Brief, risk-communication counselling that links latent infection to future disease can shift perceptions from complacency to prevention. User-friendly information—delivered through leaflets, chatbots or sequenced text reminders—reinforces the message between visits. Expanding access to short-course, once-weekly or dispersible paediatric rifapentine-based regimens reduces pill burden and fear of toxicity, while continuous medical-education and bedside mentoring reassure clinicians that preventive therapy is both effective and unlikely to drive resistance.

### Interpersonal level

Community storytelling campaigns featuring peers who have completed TPT can normalise preventive care and chip away at stigma. Secure tele-consent platforms enable migrant parents to grant legally recognised authorisation from abroad, ensuring that children can start therapy without delay.

### Institutional level

Regular in-service mentoring, searchable repositories of standard operating procedures and deliberate staff-retention or rotation schemes protect institutional knowledge. Electronic logistics-management systems with buffer-stock alerts prevent stock-outs, and digital adherence technologies—smart pill boxes, short message services check-ins or electronic directly observed therapy—provide programme managers with real-time data to target support when doses are missed.

### Community level

Decentralising TPT initiation and refills to primary-care posts, local pharmacies and trained community-health workers places services within reach of most households. Periodic “one-stop-shop” outreach days that bundle screening, chest radiography, counselling and drug pick-up into a single encounter—supplemented by mobile clinics or transport vouchers—further reduce the logistical burden. Mapping service gaps guides where and when outreach is deployed.

### Policy level

Fast-track registration and procurement of child-friendly fixed-dose combinations; embed the latest WHO recommendations in supervisory tools, quality standards and procurement specifications; and adopt ICD-10 coding to make TB infection notifiable. Reduce donor dependence through a phased financing transition: create dedicated domestic budget lines for TPT (drugs, diagnostics, contact investigation), integrate TPT into national health-insurance benefit packages with clear reimbursement rules, and align costs within medium-term expenditure frameworks. Strengthen value for money using pooled procurement and price negotiations, framework contracts and, where appropriate, regional joint purchasing. Issue clear regulatory circulars that authorise preventive regimens (including defined off-label use), standardise pharmacovigilance reporting, indemnify prescribers, and require routine safety and completion indicators to be reported alongside coverage.

### Cross-cutting measures

Integrating TPT into HIV clinics, antenatal care, diabetes services and routine primary-health-care visits repositions prevention as an everyday part of chronic-disease management. Decentralisation ensures equitable geographic coverage, and guideline-adaptation workshops tailor global standards to local realities. A multisector task force—bringing together national TB programmes, finance ministries, professional associations and civil-society organisations—can synchronise resource mobilisation, price negotiation and progress monitoring, ensuring that each layer of the health system pulls in the same direction.

## Conclusions

Our situational analysis confirms that TPT can be scaled quickly—short-course regimens are now available in most countries and overall enrolment has risen—yet performance remains inconsistent across age groups, risk categories and stages of the care cascade. Coverage is highest for adults living with HIV and for household contacts under five, but lags for older household contacts; initiation and completion rates also drop sharply after screening. Key bottlenecks include limited paediatric formulations, reliance on donor funding, stock-outs, stigma, and regulatory ambiguity around preventive regimens.

The findings translate into a practical agenda that national and sub-national programmes can adapt. At delivery level, integrate TPT assessment and initiation into existing HIV, maternal and primary-care visits; schedule periodic “one-stop” outreach days for screening, counselling and drug pick-up; and standardise a default short-course regimen with simple job-aids. District managers can track six indicators—contacts screened, initiation among those eligible, completion among those initiated, share on short regimens, days of stock-out and adverse-event reports—to pinpoint gaps and course-correct. At policy level, countries should fast-track child-friendly fixed-dose combinations, adopt ICD-10 coding to make TB infection notifiable, and phase donor dependence into domestic and health-insurance budgets using pooled procurement to cut prices. Implemented together, these actions can turn TPT from a guideline on paper into a routine, life-saving service throughout the Western Pacific Region.

## Supplementary Information

Additional file1 (DOCX 623 kb)

## Data Availability

No datasets were generated or analysed during the current study.
