# Supplementary material for: Progress and challenges in tuberculosis preventive treatment in the Western Pacific Region: a situational analysis of seven high tuberculosis burden countries
Source: Trop Med Health. 2025 Sep 2;53:122. doi: 10.1186/s41182-025-00805-6 (PMC12403340; doi:10.1186/s41182-025-00805-6)
Supplement: Supplementary file 1 — Additional file1 (DOCX 623 kb) [file 41182_2025_805_MOESM1_ESM.docx]

**Progress and challenges in tuberculosis preventive treatment in the Western Pacific Region: a situational analysis of seven high tuberculosis burden countries**

Kyung Hyun Oh^1*^, Alvin Kuo Jing Teo^2,3,4^, Manami Yanagawa^1^, Avinash Kanchar^5^, Dennis Falzon^5^, Cecily Miller^5^, Youngeun Choi^6^, Gyeong In Lee^6^, Fukushi Morishita^1^, Kalpeshsinh Rahevar^1^, Huong Thi Giang Tran^1^, Rajendra Prasad Hubraj Yadav^1^, for the Regional TB Consortium^**^

1. World Health Organization Regional Office for the Western Pacific, Manila, Philippines
2. Faculty of Medicine and Health, University of Sydney, NSW, Australia
3. The University of Sydney Infectious Diseases Institute (Sydney ID), NSW, Australia
4. Saw Swee Hock School of Public Health, National University of Singapore and National University Health System, Singapore
5. World Health Organization, Global Programme on Tuberculosis & Lung Health, Geneva, Switzerland
6. Korean Institute of Tuberculosis, Korean National Tuberculosis Association, Cheongju, Republic of Korea

*Corresponding author

World Health Organization Regional Office for the Western Pacific, Manila, Philippines.

Tel: +63 2 5289708; [ohk@who.int](mailto:ohk@who.int)

**Supplementary Figures (below)**


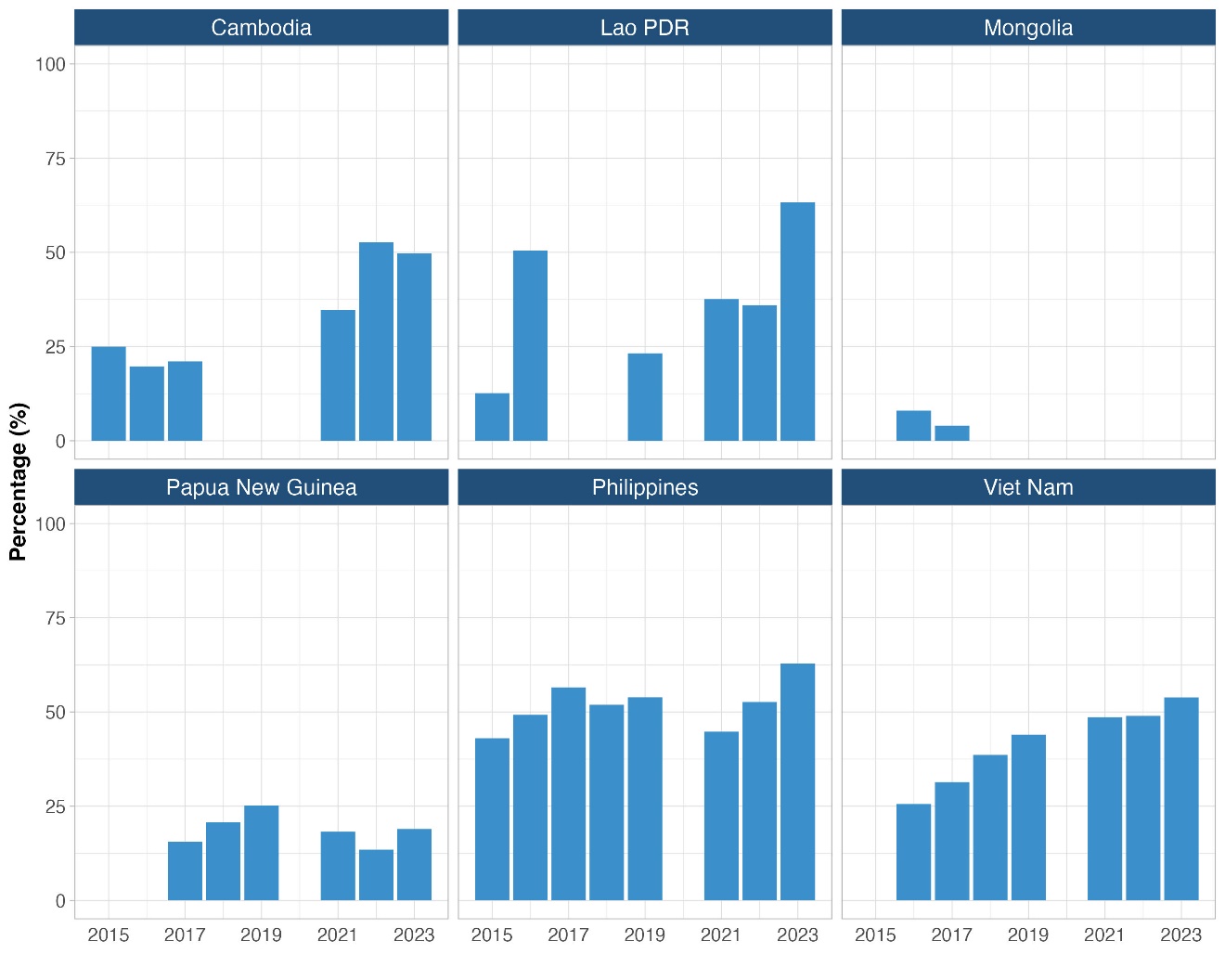


Figure S1. Percentage of tuberculosis preventive treatment among people living with HIV in six priority countries of the Western Pacific Region, 2015-2023


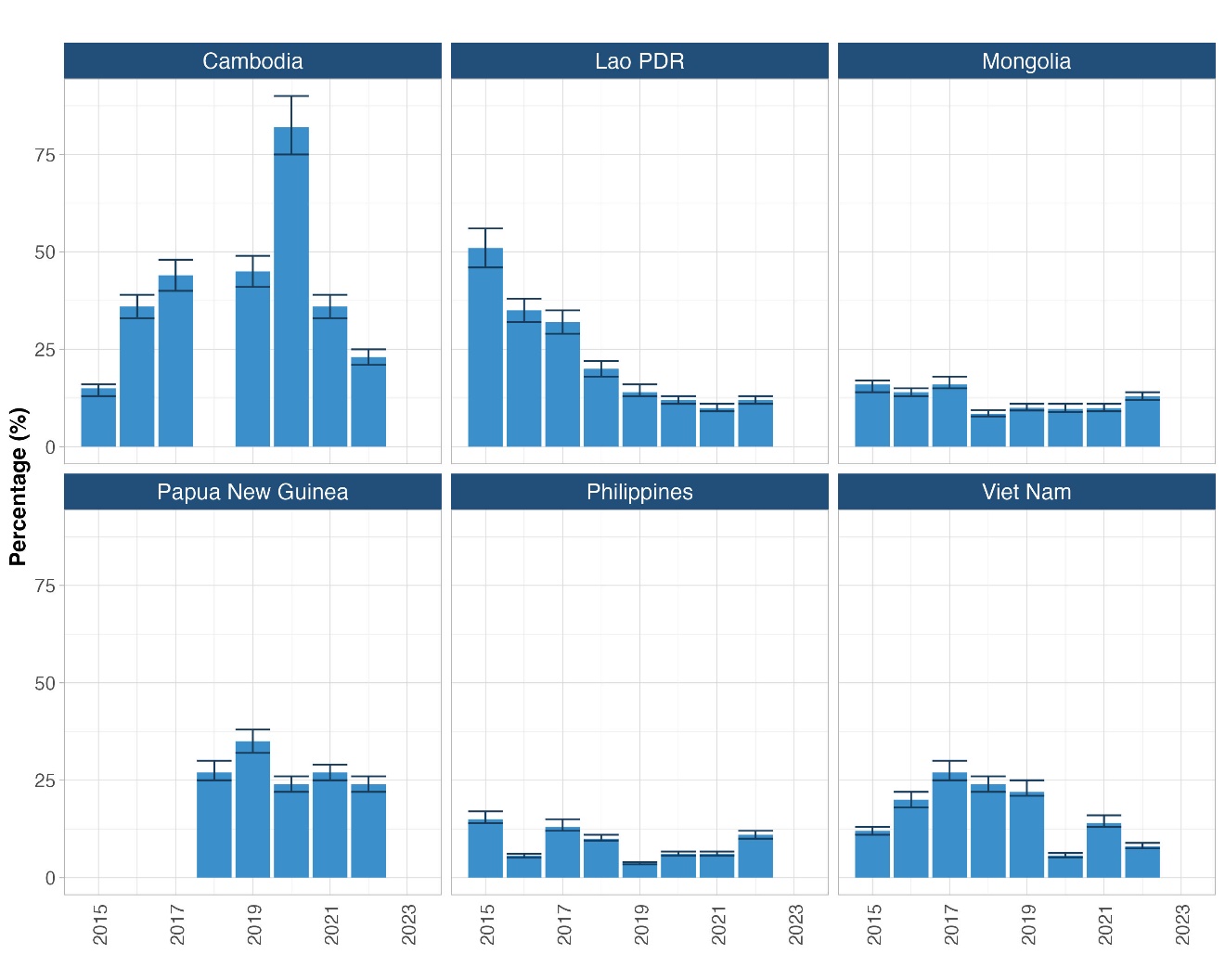


Figure S2. Percentage of tuberculosis preventive treatment among household contacts aged under five years in six priority countries of the Western Pacific Region, 2015-2023


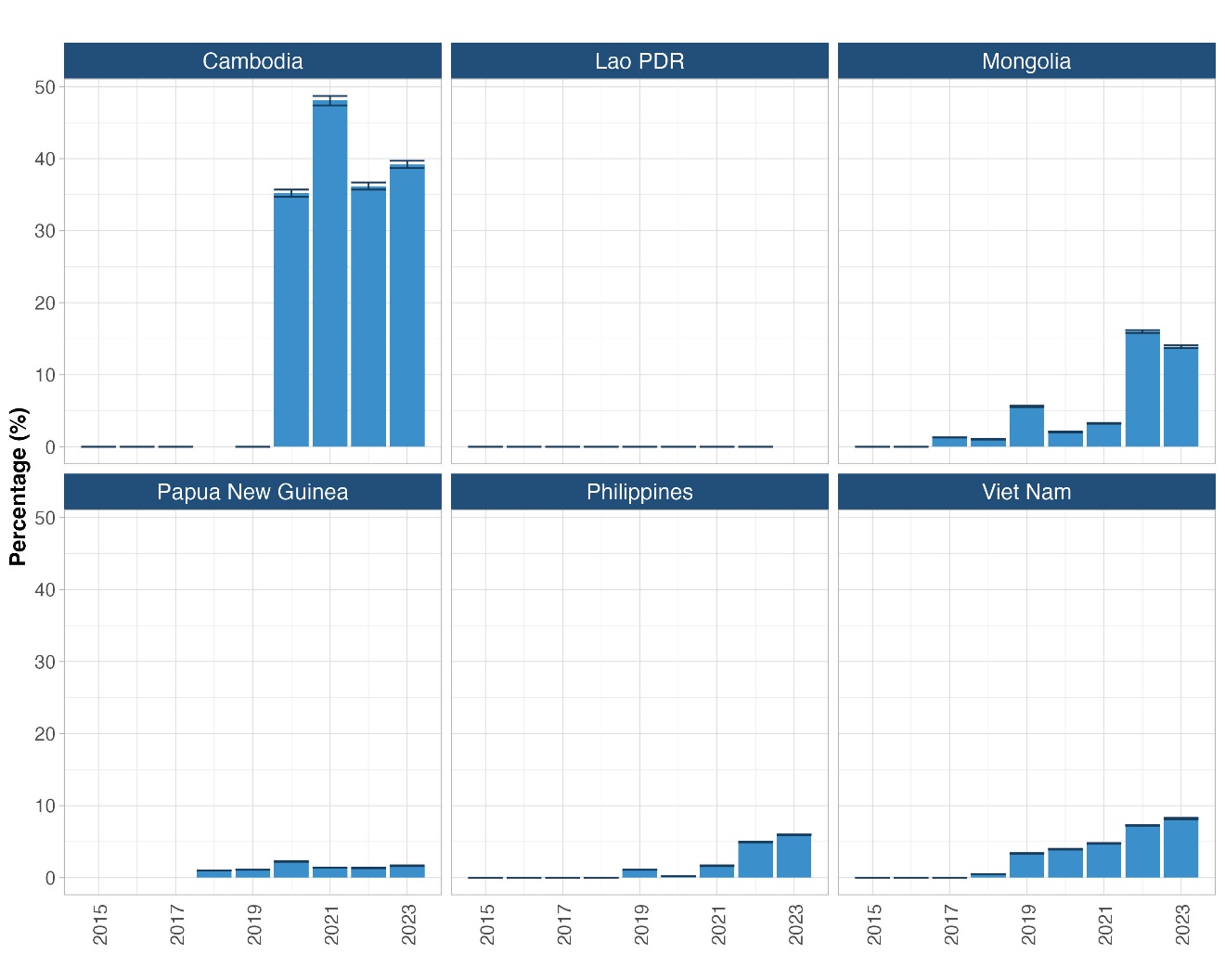


Figure S3. Percentage of tuberculosis preventive treatment among household contacts aged five years and over in six priority countries of the Western Pacific Region, 2015-2023
